# Specificity Influences in (1→3)-β-d-Glucan-Supported Diagnosis of Invasive Fungal Disease

**DOI:** 10.3390/jof7010014

**Published:** 2020-12-29

**Authors:** Malcolm A. Finkelman

**Affiliations:** Associates of Cape Cod, Inc., 124 Bernard E. St. Jean Drive, East Falmouth, MA 02536, USA; MFinkelman@acciusa.com

**Keywords:** beta-glucan, diagnostic, fungal, contamination, parenteral, bacterial

## Abstract

(1→3)-β-glucan (BDG) testing as an adjunct in the diagnosis of invasive fungal disease (IFD) has been in use for nearly three decades. While BDG has a very high negative predictive value in this setting, diagnostic false positives may occur, limiting specificity and positive predictive value. Although results may be diagnostically false positive, they are analytically correct, due to the presence of BDG in the circulation. This review surveys the non-IFD causes of elevated circulating BDG. These are in the main, iatrogenic patient contamination through the use of BDG-containing medical devices and parenterally-delivered materials as well as translocation of intestinal luminal BDG due to mucosal barrier injury. Additionally, infection with *Nocardia* sp. may also contribute to elevated circulating BDG. Knowledge of the factors which may contribute to such non-IFD-related test results can improve the planning and interpretation of BDG assays and permit investigational strategies, such as serial sampling and BDG clearance evaluation, to assess the likelihood of contamination and improve patient care.

## 1. Introduction

Over the last four decades, increasing numbers of fungal biomarkers have been added to the roster of tests available as aids to the diagnosis of invasive fungal disease (IFD) [1,2,3]. Among these is (1→3)-β-glucan, a cell wall component of almost all pathogenic fungi, with the exception of the *Mucorales* [4,5]. At this point, analysis of circulating titers of (1→3)-β-d-glucan (BDG) has been practiced for almost three decades, as an adjunct to the diagnosis of IFD [6]. The BDG test offers a relatively simple, non-invasive opportunity to obtain information on a near pan-fungal biomarker with demonstrated relevance to IFD diagnosis and, importantly, anti-fungal stewardship [7,8]. As such, over the years, it has been incorporated into an increasing number of clinical guidelines and routine practice algorithms [9,10].

**Figure 1:**
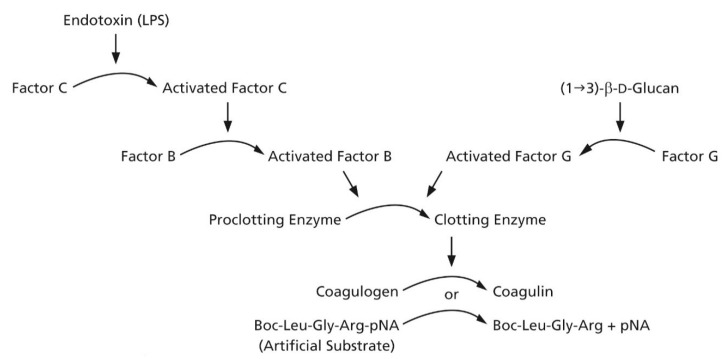
*Limulus* amebocyte lysate cascade.

Commercialization of BDG diagnostics has primarily involved reagent preparations from the blood cells of two genera of Horseshoe Crabs, *Limulus polyphemus* and *Tachypleus tridentatus* [11,12]. The former is found only on the east coast of North America while the latter is found off the coast of China and in coastal waters to its south. These preparations of BDG detection reagents utilize a protein zymogen extract from Horseshoe Crab granulocytes commonly referred to as *Limulus* amebocyte lysate or LAL [13]. The key detection components are referred to as the LAL cascade. The LAL cascade is comprised of two independent proteolytic activation pathways, one activated by bacterial endotoxin and the other by BDG, and a common terminal protease (Figure 1) [11].

**Figure 2:**
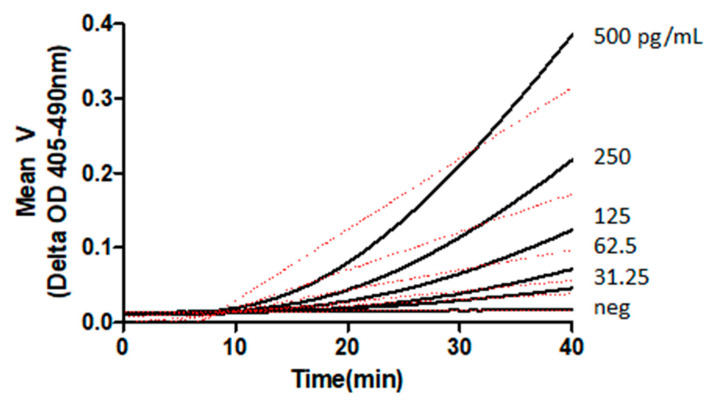
Kinetic analysis of BDG activation of LAL cascade [Unpublished data, Associates of Cape Cod, Inc.]. BDG: (1→3)-β-glucan; LAL: *Limulus* amebocyte lysate.

Inactivation, or elimination, of the endotoxin-specific components of the cascade renders it exclusively sensitive to BDG, permitting the creation of a reagent suitable for laboratory assays for BDG [14,15]. These assays have been established, commercially, using kinetic photometric methods [16]. Examples of chromogenic kinetic reactions of BDG are presented in Figure 2. The linear range of glucan concentrations accessible to LAL-based reagent BDG detection is dependent upon the formulation used and normally extends over a serum range that includes a negative, indeterminate, and positive range, with respect to invasive fungal disease [17].

## 2. (1→3)-β-Glucan Distribution and Structure

**Figure 3:**
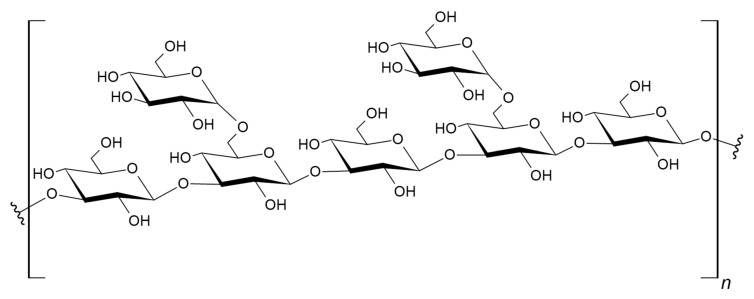
Fungal BDG: (1→3)-β-glucan backbone with (1→6)-β-linked glucose.

(1→3)-β-glucan is widely synthesized, for multiple purposes, among bacteria, fungi, algae, and plants [18,19,20,21]. It is not produced in mammals, nor are mammals capable of its enzymatic degradation [22]. Degradation of BDG has been observed in phagocytic cells and is presumed to occur chemically, through hydrolytic mechanisms [23]. Some of the myriad functions of BDG include use as a cell wall structural material in fungi [24], as extra-cellular and intra-cellular matrix material in certain bacteria [25], and algae [26], respectively. In plants, it appears as a wound repair and specialized structural material [27], typically in wound response tissue [28], vascular elements [29], and as intracellular pressure regulators in plasmodesmata [30]. In mammals and insects, which do not make BDG, it is recognized as an innate immune system-activating pathogen-associated molecular pattern and influences innate immuno-metabolism [31,32,33]. BDG is comprised of sequential d-glucose molecules linked by (1→3)-β-glycosidic linkages. The primary backbone structure of BDG is presented in Figure 3, with a fungi-typical (1→6)-β-linked glucose branch. The β linkage imposes steric constraints upon the orientation of the adjacent glucose molecules leading to the formation of a helical structure as the polyglucan chain becomes elongated [34,35,36]. Side chain addition, or branching, in multiple forms and with multiple moieties is also observed to occur, creating characteristic BDG types among different taxonomic groups [37]. Single helical BDG is observed to anneal through hydrogen bonding and hydrophobic interactions, resulting in native triple helical structures. Assemblages of triple helical structures also form, leading to ordered cable-like materials that contribute mechanical strength and structure to fungal cell walls [38].

## 3. Diagnostic Performance and the Role of Non-IFD Origin BDG 

BDG can be contributed to the circulation during cell wall remodeling in the life cycle of fungal cells [39]. The extent to which this occurs is dependent upon myriad factors including fungal genus and species, site of infection, access to the vasculature, host factors including immune response, and clearance efficacy. Assay reactivity is dependent upon structural factors such as BDG molecular weight, branching, and single versus triple helical structure [40]. While commercial BDG assays are considered qualitative assays, due to the multiple factors that can contribute to titer, they have continuous numerical outputs which have permitted the validation of clinical thresholds for negative, indeterminate, and positive association with invasive fungal disease. In this context, the results of BDG testing are usually evaluated using the principles appropriate to quantitative assays [7].

The diagnostic performance of BDG assays has been assessed in approximately 200 publications, including multiple meta-analyses [7,41]. Although the heterogeneity of many studies is relatively high, diagnostic performance characteristics are reasonably reproducible. Typically, BDG negative predictive value is very high, often greater than 95%, while positive predictive value is lower, due to observations of BDG titers above the positive threshold, which are deemed to be diagnostic false positives [42]. Analytically, the BDG reagents are being activated by the actual presence of BDG in the serum samples. Accordingly, these findings beg the question of the source or sources of the BDG. At this point, three decades of clinical BDG testing experience as well as pre-clinical research suggest that three main sources of circulating BDG exist. These include IFD, iatrogenic contamination, and intestinal translocation. Non-specific pulmonary translocation due to fungal colonization is also a potential source, given the high levels of serum BDG observed to translocate from the alveoli in pneumocystosis [43], but non-IFD pulmonary translocation studies have not been performed and represent an area where data are needed [44].

## 4. Major Sources of Circulating BDG

### 4.1. Invasive Fungal Disease

The subject of fungal cell wall-originating BDG contribution to the circulation has been well described in numerous publications, including multiple meta-analyses [43,45,46,47,48]. This occurs as a result of the normal processes of the fungal life cycle in which the cyclical processes of wall polysaccharide synthesis and degradation occur [38,49]. Minute quantities of wall material, including BDG, are sloughed into the peri-fungal environment and migrate to the circulation. In the case of fungemia, in which the fungi are growing in the bloodstream, the contribution to the circulation is direct. The longitudinal characterization of circulating BDG titers typically demonstrates rising BDG levels early in infection, followed by slow declines which may only return to baseline well after successful disease resolution [50,51]. Another route of entry to the circulation was described by Hong et al. 2004, among others, in which phagocytosed fungal cells are degraded by hydrolytic processes within the phagolysosomes followed by externalization of degraded material [23]. BDG from phagocytosed yeast sacculi was demonstrated to be processed by macrophages both in vivo and in vitro. In the latter experiments, externalized BDG titers rose in the cell culture supernatants while declining within the cells. This may be a process that influences the observed slow decline of circulating BDG post-therapeutic success [52].

### 4.2. Iatrogenic Contamination

**Figure 4:**
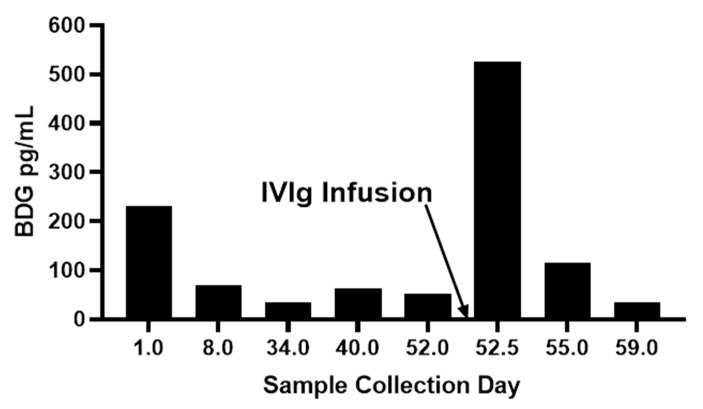
Pre-and post IVg infusion effect upon serum BDG titer [Unpublished data, Associates of Cape Cod, Inc.].

Medical treatment-related BDG contamination has been assessed in a variety of clinical contexts including multiple types of intravenously administered materials including drugs and blood fractionation products, invasive use of surgical materials, and cellulosic dialysis membranes Table 1. In the case of drug formulations, BDG may be present in the original source material itself, such as products made by fungal fermentation [53], in excipients added to the formulation [54], from media used in microbial or cell culture [55], or from process equipment, materials, and solutions [56]. In the case of blood fractionation products such as IVIg and serum albumin, the filtration of blood plasma through cellulosic depth filters can result in leached plant BDG (callose) [57,58]. It is fairly common to observe highly elevated BDG titers in patients receiving albumin or immunoglobulin (IVIg) infusions [59]. These high titers are usually observed to decline relatively quickly and such responses support suspicion of iatrogenic contamination. Figure 4 present observations for serum BDG pre-and post-infusion of an intravenous immunoglobulin product and the follow-up titers from subsequent blood draws two days and a week later. The very rapid rise and fall of serum BDG subsequent to its infusion is characteristic of such contamination events.

Surgical materials such as sponges and gauze permit large quantities of BDG (up to millions of pg/gm of gauze or sponge material) to be leached during intra-cavity or other invasive use, as well as contact with surgical solutions and bodily fluids such as with certain autologous blood recovery procedures [60,61]. Mohr et al. 2011, demonstrated that BDG diagnostic specificity in post-surgical patients improved, substantially, between sampling immediately post-surgery and after 3 days [62]. Similarly, the use of gauze in surgery has been observed to contribute to leaching and elevated circulating burdens of BDG [63]. BDG is also a licensed drug in certain countries, as an anticancer adjunctive therapy based upon its innate immune activation properties. Injected particulate BDG has been shown to generate elevated circulatory BDG for years after administration, making detailed medical history analysis critical to interpretation of unexpectedly high BDG titers in patients in whom IFD is unsuspected [64]. Renal replacement therapy utilizing regenerated cellulose was demonstrated to contribute to elevated BDG in hemodialysis patients [65]. As regenerated cellulose dialysis membranes have been replaced by non-BDG-leaching synthetic membranes, these are now an unlikely source [66]. 

Based upon these observations of patient contamination by multiple types of medical materials and procedures, blood draw planning for BDG titer determination should take these into account and unexpected BDG elevation should prompt chart review. 

Another category of iatrogenic patient contamination, one that has had significant debate, is that of parenteral administration of potentially contaminated antibiotics. Multiple publications asserting the occurrence of patient false positives by this route as well as the opposite have been presented [67,68,69]. While this route of patient contamination is possible, the high level of dilution generated upon injection of relatively low volumes of antibiotic make this unlikely. Further, the high negative predictive value for IFD observed for patients receiving a vast array of antibiotics suggests that this is not a significant problem [70,71].

### 4.3. Intestinal Translocation

The past two decades have seen an explosion in data regarding intestinal barrier permeability, its myriad causes, and its relationship to inflammation-related pathophysiology [73,74,75]. Multiple biomarkers such as zonulin and various claudins have been identified as useful markers in characterizing the nature of barrier injury [76,77]. Data regarding elevated circulating BDG, its correlation with other markers of microbial translocation and inflammation, and orthogonal testing with enteral polysaccharides such as fluorescein labeled dextran demonstrate that it is also a translocated entity [78,79,80]. Ellis et al. 2008 observed that in a series of non-IFD hematological malignancy patients undergoing chemotherapy, those with enterocyte damage and/or mucositis had BDG titers that were persistently elevated compared to patients without those pathologies (*p* = 0.002) [78]. MAF: Sentence added with a new reference to restore numerical reference sequencing. Pre-clinical models of sepsis also demonstrate elevated levels of circulating BDG [79]. The kinds of insults that contribute to intestinal barrier permeability include intestinal ischemia, mesenteric hypoxia, microbial toxins, viral infection of intestinal tissue, metabolic toxicity such as in uremia, chemotherapy-associated mucositis, large total surface area burns, and protease-producing intestinal enterococci (Table 2). All of these conditions have been observed to be associated with elevated BDG titers. Thus, it is important to fully understand the host and clinical factors that could be contributing to intestinal barrier permeability as part of the consideration of BDG titer interpretation.

Several conditions may contribute to intestinal hypoxia, which is a causative factor in intestinal permeability barrier injury. These include large surface area burns, intestinal ischemia reperfusion injury, mesenteric hypoxia, and intra-dialytic hypotension. Studies have described the relationship between large surface area burns and BDG false positives [81,92]. Similarly, a Japanese study evaluated the use of BDG testing in the diagnosis of candidemia in severe burn patients and demonstrated high sensitivity, 100%, but lower specificity, 68%, when the test manufacturer’s positive cutoff of 11 pg/mL was used. A cutoff of 40 pg/mL generated a sensitivity of 100% with a specificity of 95% [93]. Validation of alternative cutoffs in specific clinical contexts known to contribute to elevated BDG titer may represent a means of dealing with specificity issues. 

End stage renal disease is a condition associated with intestinal permeability barrier injury and microbial metabolite translocation from the lumen [94]. Additionally, hemodialysis is associated with intradialytic hypotension and attendant loss of intestinal permeability barrier patency [95]. It has been reported that hemodialysis within the previous 72 h was associated with BDG false positives, *p* = 0.011 [96]. 

### 4.4. Intestinal Contents: Mycobiome and BDG Translocation

Translocation of BDG from the intestinal lumen through the intestinal epithelium and then to the wider circulation raises questions concerning the sources of luminal BDG. Research over the last few decades suggests that both ingested foodstuffs and the intestinal mycobiome are potential contributors. With respect to the former, certain foods are extremely rich in BDG. Oat bran BDG content is in the range of 4.5%, by weight [97]. Similarly, foods rich in fungal mycelium, such as mushrooms, represent potential sources of BDG which might translocate. At this point, the case for translocating foodstuff-derived BDG remains to be more fully evaluated. A recent study evaluated a single bolus of extremely high BDG content foodstuff consumption in an HIV population and serial testing over 24 h failed to reveal meaningful serum BDG titer elevation [98]. The intestinal mycobiome represents another potential source of translocatable BDG and there are structural differences between fungal and plant-origin BDG which may affect translocation. This is of particular interest in patients receiving gut-active broad spectrum antibacterials. The administration of such drugs has been demonstrated to produce 1–2.5 log increases in gut *Candida* populations in patients [99]. It is possible that the release of mycobiome-origin BDG in the gut lumen, proximal to the luminal epithelium, may result in more efficient translocation. These are areas of continuing investigation with the potential to illuminate prognosis of gut-originating candidiasis and inflammatory disease. In this light, recently published data by Zhai et al. 2020 revealed an expansion of pathogenic *Candida* species in the intestinal mycobiota of post-stem cell transplant patients two–ten days prior to their developing candidemia. DNA analysis showed that the candidemia strains clustered tightly with the luminal contents strains [100]. Similarly, the role of the gut mycobiome has been examined in the context of alcohol-induced liver disease in a chimeric murine model. In this model, alcohol feeding resulted in intestinal *Candida* overgrowth and BDG translocation with resulting hepatic inflammation, thought to occur through BDG ligation of hepatic Kuppfer cell dectin-1. Dectin-1 knock-out mice failed to develop the inflammation, leading to the conclusion that translocated intestinal origin BDG played a major role [101]. 

### 4.5. Hepatic Function

In animal models, BDG is primarily cleared through the liver [102]. Human clinical conditions which result in reduced hepatic function have been shown to be associated with elevated serum BDG. Sanada et al. 2014 have described the relationship between serum BDG levels in the setting of pediatric end stage liver disease [103]. The liver clearance function was underscored by significantly higher BDG titers in portal blood relative to the peripheral circulation. There was a very significant negative correlation (*p* < 0.001) between the pediatric end-stage liver disease patients score and the hepatic clearance of BDG in the transplant patients. Sanada et al. 2012 also described elevated serum BDG in end-stage liver disease patients with and without fungal infections. Of note, median hepatic clearance of circulating BDG was measured as 87.9% [104]. Elevated patient peripheral blood levels of BDG pre-transplant predicted a much longer course of recovery (*p* < 0.001) and, post-operatively, fungal infections. The peripheral BDG titers in the infected patients were significantly higher than the uninfected patient levels, suggesting the potential of diagnostic utility despite compromised clearance. Recently, Moon et al. 2020 analyzed microbial translocation markers in the blood of hepatitis C virus infected patients [85]. Serum BDG was significantly elevated in hepatic fibrosis (Ishak Score 0–2 and 5–6), relative to controls (Mann–Whitney, *p* < 0.0001 and *p* < 0.001, respectively). That this may be due to inadequate clearance rather than intestinal translocation is suggested by observations that enhanced microbial translocation was not observed in HCV-infected chimpanzees which do not develop liver fibrosis [105]. Thus, hepatic function status is an additional factor to be considered in the evaluation of the significance of circulating BDG titers.

### 4.6. Bacterial Infections

As discussed earlier in this review, a number of publications have asserted that bacterial infections have been the source of elevated serum BDG [106]. While elevated BDG titers have been associated with bacterial infections in a number of studies, the demonstration of a contribution of BDG derived directly from the bacterium has not, in the main, been systematically demonstrated. Mennink-Kersten and colleagues described elevated BDG titers in a case series of bacteremic patients, particularly in infections with multiple *Pseudomonas* sp. and a *Streptococcus pneumoniae* [107]. Among the *Streptococci*, *S. pneumoniae* Type 37 has been described as producing a BDG with a (1→3)-β-backbone with each glucose moiety having a (1→2)-β-glucose side chain [108]. Held et al. 2013 described a mean serum BDG titer of 135 pg/mL in patients with enterococcemia while patients with other causes of bacteremia displayed a mean level of 15 pg/mL [84]. Enterococcal species, which produce exopolysaccharides of mixed monosaccharide and glycosidic linkage composition, are not known to produce BDG. As the etiology of enterococcal bacteremia includes overgrowth in the intestinal lumen followed by invasion through the intestinal barrier [109], the possibility of BDG translocation through a damaged intestinal barrier represents a plausible mechanism of its elevation in the circulation. Recently, the Enterococcal exopolysaccharide poly-*N*-acetyl glucosamine has been determined to be a virulence factor due to its role in breaching the intestinal permeability barrier [109]. In contrast to bacterial species not observed to directly produce BDG, circulating BDG derived from invasive *Nocardia* infections has been definitively demonstrated in a series of independent case reports. Elevated BDG titers were reported in infections caused by multiple species of *Nocardia* including *N.abscessus*, *N. elegans*, *N. farcinica*, and *N. nova* [110,111]. Additional evaluation to verify the presence of BDG involving the culturing of clinical isolates of *N. asteroides*, *N. neocalidoniensis*, and *N. cyriacgeorgica*, as well as the control species *Staphylococcus aureus* and *Escherichia coli*, and the semi-purification of the post-growth culture broth supernatants was conducted. The un-inoculated medium and the controls were negative for BDG while the *Nocardia* species’ supernatants were all over-range in the assay, confirming the source of the BDG as the *Nocardia* infections [112]. Thus, while a number of bacterial genera are known to produce BDG, very few are human pathogens. Of those pathogenic genera that do make beta-linked polyglucans, the genus *Pseudomonas* makes small, 2-12-mer, periplasmic osmoregulatory cyclic (1→2)-β-linked glucan sequences [113] which are broadly similar to the short (5-12-mer) branched (1→2)-β-linked glucans produced by *E. coli* [114]. Accordingly, while some bacteria are capable of producing (1→3)-β-linked beta-glucans, such as *Agrobacterium tumefaciens* [115], of those genera that are human pathogens it is rare that they make the (1→3)-β-linked glucan structures that are the only forms known to be capable of the activation of *Limulus*-based detection reagents.

## 5. Manufacturing-Associated BDG Contamination

Production processes for parenterals are another potential source of patient-contaminating BDG. During manufacturing, the introduction of BDG to parenterals may occur through multiple sources. These include fermentation media components such as yeast extracts, plant material extracts, sugars, as well as the use of fungal organisms as the source of fermentation products [53,55,57]. If downstream processing does not remove the contaminating BDG, it may result in patient exposure. Examples of patient contamination after infusions have been reported in the literature and represent a failure to control an easily measurable contaminant [59,116]. Another source of parenteral contamination is the use of cellulosic depth filters [57]. These devices are generally mixtures of cellulose and diatomaceous earth and are used to provide initial clarification of cell culture fluid, microbial fermentation broths, and blood plasma. As plant material contains small quantities of BDG (callose) in various plant tissues [117], BDG is readily leached from the depth filters to enter downstream processing as a contaminant [57,118], Blood fractionation products, whose starting material is depth filtered blood plasma, are recognized sources of product contamination with BDG [119,120]. 

In addition to process equipment, process solutions may contain BDG and introduce contamination. Vigor et al. 2017 reported two sources of process-related BDG contamination. One was BDG contamination in sucrose, used as a formulating excipient, and the other was a virus filter storage solution [56]. With its recognition as a bioactive contaminant, pharmaceutical manufacturing organizations are beginning to measure and control BDG contamination of parenterals [55,57].

## 6. False Positive Investigation

BDG is indicated as an adjunct to the diagnosis of invasive fungal disease. It is to be considered with reference to host, clinical, and other laboratory characteristics and findings. When an elevated BDG titer appears to be discordant with respect to these other observations, it is useful to evaluate potential sources of diagnostically false positive BDG presence. Table 3 provides a listing of a series of medical product and clinical condition-related questions which may help to clarify a previously unrecognized source of non-IFD-related BDG.

In addition to investigation of the above-listed potential sources of circulating BDG, support for the likely presence of a fungal source of the observed BDG may be found by utilizing additional tests. These include microscopic examination of appropriate patient tissue and fluid samples, as well as various PCR and antigen tests, as well as emerging novel diagnostic technologies [121]. Positive results on ancillary tests for the presence of fungi can add to support for ruling out BDG diagnostic false positivity. Multiple studies have reported improved specificity in the setting of multiple fungal marker utilization. Boch et al. 2016 reported the increase in specificity, in the setting of invasive aspergillosis, from 48% to 94% when serum BDG results were combined with BAL galactomannan results [122]. In the setting of acute leukemia, Qian et al. 2019 described specificity values for either BDG or GM alone, for invasive fungal disease, at 90% and 40%, respectively [123]. In combination, the specificity was 98%. Similarly, Urabe et al. 2017 demonstrated that combining *Aspergillus* PCR with BDG raised specificity from 48.2% in bronchoalveolar lavage fluid, to 96.7% and 99.2% with two different PCR assays, respectively [124]. In contrast, Held et al. 2013 reported that, in the setting of candidemia, combining mannan antigen testing with serum BDG lowered the specificity marginally, from 85.5% with BDG alone to 85.0% [84].

## 7. Discussion

As described above, circulating BDG titer is an important adjunct in the diagnostic process for patients suspected of invasive fungal infections. While the NPV is very high, optimizing the utility of positive BDG results requires consideration of both host and clinical factors, as well as potential sources of iatrogenic contamination. The latter factors may include introduction of BDG-contaminated materials in the course of medical care. In that circumstance, institutional observations suggesting an association between elevated BDG and certain parenterals or devices should prompt evaluation of BDG burdens present in those products. Prospective BDG testing practices should employ blood draws taken prior to introduction of materials considered suspect. Similarly, blood draws obtained during the initial 3 days post-surgery involving intra-cavity use of gauze and surgical sponges need to be interpreted cautiously and the observation of very rapidly declining serial sample values may indicate contamination rather than infection. In addition, BDG testing results of samples taken after intra-operative autologous blood recovery using surgical sponges must be similarly considered. In patients with intestinal permeability barrier injury risk factors, the presence of elevated BDG absent a diagnosis of IFD and iatrogenic contamination should prompt consideration of potential luminal contents translocation. This is also of significant importance given the association of intestinal permeability barrier injury and the potential for infection due to translocation of viable microorganisms, including *Candida*, to the circulation. Other potential sources of elevated BDG include hepatic insufficiency, potentially leading to reduced BDG clearance, and infection with *Nocardiales* sp. 

Invasive fungal disease continues to have high morbidity and mortality and the development of adequate diagnostics has been challenging [3,125]. Fungal antigen tests, including BDG, represent advances toward the goal of improving the effective diagnosis of IFD. In order for them to be used effectively, it is critical that laboratories and health care practitioners are well acquainted with the factors that can influence results, within the patient’s clinical context.

## Figures and Tables

**Table 1:** Medical products and processes associated with patient/product contamination.

Material	Reference
Gauze [63]	Kanamori, H. *Tohoku J. Exp. Med.* 2009, *217*, 117–121.
Surgical sponge [60]	Stycznski, A. *J. Fungi (Basel)* 2018, *4*, E114.
Process equipment [56]	Vigor, K. *Biotechnol. Prog.* 2016, *32*, 1494–1502
Fermentation media [58]	Gefroh, E. *Biotechnol. Prog.* 2013, *29*, 672–680.
Cellulosic depth filters [57]	Holstein, M. *Biotechnol. Prog.* 2020, e3086
Hemodialysis with cellulosic membranes [65]	Kanda, H. *Kidney Int.* 2001, *60*, 319–323.
Intravenous immunoglobulin [52,59]	Angebault, C. *Open Forum Infect. Dis.* 2016, *3*, ofw128.Duffner, U. *Bone Marrow Transpl.* 2012, *47*, 151–152.
Human serum albumin [72]	Nakae, H. *Acute Med. Surg.* 2017, *4*, 251–254
Anti-tumor adjuvant [64]	Tokuyasu, U.H. *Int. J. Gen. Med.* 2010, *3*, 273–277.
Antibiotics [67,68]	Liss, B. *J. Antimicrob. Chemother.* 2016, *71*, 913–915.Furfaro, E. *Clin. Vaccine Immunol.* 2014, *21*, 1357–1359.

**Table 2:** Conditions associated with elevated circulating BDG.

Clinical Setting	Reference
Burns, large surface area [81]	Shupp, J. *Mycoses* 2011, *55*, 224–227.
Chronic kidney disease [82]	Wong, J. *BMC Nephrol.* 2020, 21, 118.
Cystic fibrosis [44]	Rautemaa, V. *DMID* 2017, *88*, 16–21.
Cytomegalovirus infection [83]	Ramendra, R. *CID* 2019, ePub.
Enteroccocemia [84]	Held, J. et al. *J. Clin. Microbiol*. **2013**, *51*, 1158–1164.
Hepatitis C virus [85]	Moon, M.S. *Open Forum Inf. Dis.* 2019, ePub.
HIV neurocognitive decline [86]	Hoenigl, M. *Medicine* 2016, *95*.
HIV non-AIDS-related adverse events [87]	Hoenigl, M. *CID* 2019, *69*, 676–686.
Invasive mechanical ventilation [88]	Heyland, D. *J. Crit. Care* 2011, *26*, 536.e1–536.e9
Lupus erythematosus [80]	Issara-Amphorn, J. *J. Innate Immun.* 2018, *10*, 18.
Sepsis-septic shock transition [89]	Leelahavanichkul, A. *Shock* 2016, *46*, 506–518.
Post-major abdom. Surgery [90]	White, P.L. *CID* 2020, In Press.
Ulcerative colitis [91]	Shah, J. *J. Dig. Dis.* 2019, *20*, 642–648.

**Table 3:** Major factors to consider in investigating suspected BDG false positives.

**A. Medical Product-Related False Positives**	
1.	Has the patient received IVIg infusions?
2.	Has the patient received human serum albumin infusions?
3.	Has the patient received total parenteral nutrition?
4.	Has the patient undergone invasive surgery within the past 4 days?
5.	Does the patient have indwelling gauze, surgical sponges packings?
6.	Are any other invasive cellulosic medical devices in use?
**B. Medical Condition-Related False Positives**	
1.	Does the patient have evidence of severe mucositis or enterocolitis?
2.	Is the patient on hemodialysis?
3.	Does the patient have invasive nocardiosis?
4.	Is gut ischemia or hypoxia suspected?
5.	Has pneumocystosis been ruled out?

## Data Availability

All non-published literature sourced data is present in the manuscript.
